# Hyperglycaemia Presenting With Hemichorea: A Rare Case of Diabetic Striatopathy With Ketosis

**DOI:** 10.7759/cureus.94931

**Published:** 2025-10-19

**Authors:** Hindol Dasgupta, Shahriar Shahjada, Janice James, Reem Abduljabbar, Asif Nawaz, Fahad Mewazy

**Affiliations:** 1 Medicine, Royal Glamorgan Hospital, Llantrisant, GBR; 2 Internal Medicine, Royal Glamorgan Hospital, Llantrisant, GBR; 3 Diabetes and Endocrinology, Royal Glamorgan Hospital, Llantrisant, GBR; 4 Radiology, Royal Glamorgan Hospital, Llantrisant, GBR

**Keywords:** chorea hyperglycemia basal ganglia syndrome, choreiform movement disorder, diabetic striatopathy, hyperglycemic hemichorea, ketotic hyperglycemia

## Abstract

Acute movement disorder is a rare complication of diabetes mellitus. Uncontrolled diabetes mellitus can present with various movement disorders, of which chorea and ballism are the most common. Various nomenclatures have been used in the past for this condition. However, diabetic striatopathy is the most common and comprehensive terminology. It refers to a condition of hyperglycaemia and an acute-onset movement disorder with or without characteristic radiological findings on CT or MRI scan. It is commonly associated with non-ketotic hyperglycaemia in the background of type 2 diabetes mellitus. Previous studies have mostly reported it in the Asian population in the sixth to seventh decades of life with a female predominance.

We present an 84-year-old British woman with type 2 diabetes mellitus, atrial fibrillation and suspected primary pancreatic malignancy, who presented after a fall and head injury with a one-week history of choreoathetoid movement of the right forearm and hand. Blood sugars and ketones were raised on presentation. CT and MRI brain demonstrated hyperdensity and T1 hyperintensity in the left basal ganglia, respectively. The patient was treated with variable-rate insulin for a prolonged period, followed by pre-mix insulin. Tetrabenazine was also started. Within one week, there was a dramatic improvement in the choreoathetoid movement.

Although increasingly recognised, diabetic striatopathy remains underreported, largely due to limited physician awareness. This case is notable for the unusual association with ketotic hyperglycaemia, advanced age and significant comorbidities. Despite these factors, the patient achieved full recovery, underscoring the importance of early recognition and symptomatic treatment in addition to metabolic correction.

Therefore, it should be considered in the list of differential diagnoses in any diabetic patient presenting with a movement disorder, even outside the typical Asian demographic and non-ketotic setting. Prompt diagnosis prevents misclassification as an intra-cerebral haemorrhage and facilitates timely management with an excellent prognosis.

## Introduction

In addition to the known neurological complications of stroke and peripheral neuropathy, uncontrolled diabetes mellitus can also lead to various acute movement disorders [1]. Diabetic striatopathy is a condition that is characterised by hyperglycaemia and chorea-ballistic movement disorder with or without characteristic radiological findings in the striatum [1-3]. It is more commonly associated with type 2 diabetes mellitus, and most cases present with non-ketotic hyperglycaemia [1-4]. Bedwell first reported hemiballismus associated with hyperglycaemia in 1960 [5]. Since then, there have been multiple case reports and a few systematic reviews; still, most physicians lack awareness of this condition [6-8]. There is a lack of clarity regarding the underlying pathophysiology of this condition [6,7,9]. It remains underdiagnosed, particularly in elderly patients, where confounding comorbidities may obscure the clinical picture [2,6]. With early diagnosis and prompt treatment, it has a very good prognosis [4,6,8,10]. However, due to the lack of awareness amongst physicians, it is often missed in the list of differential diagnoses in cases presenting with acute movement disorders, thereby leading to delayed diagnosis [6].

In this case report, we will discuss an elderly female patient presenting with hemichorea along with ketotic hyperglycaemia and characteristic basal ganglia hyperdensity on the CT scan. This case is notable due to her advanced age, coexisting comorbidities, including atrial fibrillation and suspected pancreatic malignancy, and still achieving complete recovery.

## Case presentation

An 84-year-old lady was brought to the hospital in August 2025 following a fall, head injury and loss of consciousness. She slipped when attempting to hold on to her walking frame, leading to a fall and injury to the right side of her head and subsequent loss of consciousness. She was found by paramedics and brought to the hospital. She also reported having abnormal involuntary movement of her right forearm and hand for one week. The movements were insidious in onset and happened during any voluntary action. They were absent at rest.

Her past medical history included: 1) type 2 diabetes mellitus on insulin, 2) atrial fibrillation on apixaban, 3) recent diagnosis of suspected primary malignancy in the body of the pancreas (not for further investigation and for best supportive care considering her age and frailty), and 4) previous giant cell arteritis with a prolonged course of steroid intake. She was diagnosed with diabetes mellitus nine years ago, and her glycaemic control has been sub-optimal during these years. However, there has been no previous admission with diabetic ketoacidosis or hyperosmolar hyperglycaemic state.

Her Glasgow Coma Score (GCS) was 15/15 when found by paramedics and in the hospital emergency department as well. General and systemic examination was unremarkable, other than involuntary choreoathetoid movement of her right forearm. Power and tone were normal in all four limbs. Sensation was intact. Cranial nerve examination was unremarkable. Her vital signs were stable (Table 1).

**Table 1:** Vital signs on presentation mm of Hg: millimetres of mercury

Vital Sign	Values
Blood Pressure	122/82 mm of Hg
Heart Rate	68 per minute
Temperature	36.8-degree celcius
Oxygen Saturation	98% on room air
Respiratory Rate	16 per minute

Initial investigations (Table 2) revealed a high blood sugar level at 21.1 mmol/L, a raised blood ketone level at 3.5 mmol/L, and a raised lactate level of 3.2 mmol/L. Her pH was 7.34, and bicarbonate was 22.8 mmol/L, thereby ruling out diabetic ketoacidosis. Her renal function test was slightly deranged with a raised creatinine level of 143 micromol/L, urea of 6.2 mmol/L, sodium of 127 mmol/L and potassium of 3.2 mmol/L, changes likely secondary to dehydration. Her CRP was slightly raised at 30 mg/L. Her HbA1C in 2024 was 73 mmol/L, indicating long-term poor glycaemic control. Based on the biochemical investigations, it is difficult to say what triggered the raised blood sugar and raised ketones. It is evident from the investigations that she was dehydrated. Otherwise, the rest of the blood investigations, including full blood count and liver function test, were unremarkable. ECG showed atrial fibrillation consistent with her known diagnosis.

**Table 2:** Initial blood investigations on presentation mmol/L=millimoles per litre, micromol/L=micromoles per litre, mg/L=milligrams per litre, mg/dl=milligrams per decilitre

Investigation	Value	Normal Reference Range
Blood Sugar	21.1 mmol/L (379.8 mg/dl)	4-11 mmol/L
Blood Ketone	3.5 mmol/L (20.33 mg/dl)	Below 0.6 mmol/L
pH	7.34	7.35-7.45
Serum Bicarbonate	22.8 mmol/L	22-26 mmol/L
Serum Lactate	3.2 mmol/L	Below 2 mmol/L
HbA1c	73 mmol/L	Below 48 mmol/L in diabetic patients
Serum Urea	6.2 mmol/L	2.5-7.8 mmol/L
Serum Creatinine	143 micromol/L	58-110 micromol/L
Serum Sodium	127 mmol/L	133-146 mmol/L
Serum Potassium	3.2 mmol/L	3.5-5.3 mmol/L
Serum C-Reactive Protein (CRP)	30 mg/L	Below 5 mg/L

**Figure 1:**
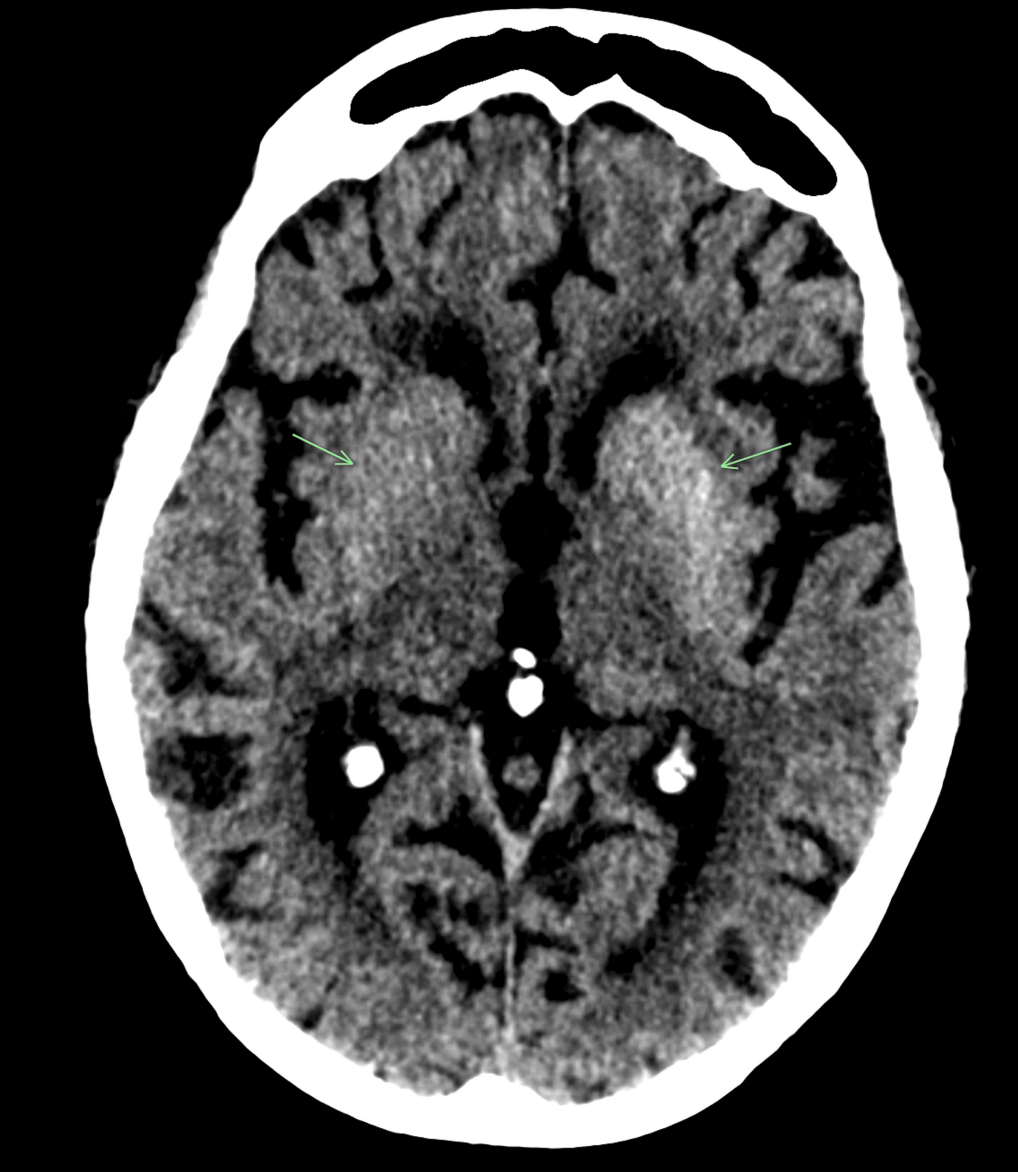
CT head showing hyperdensity in the region of the left basal ganglia Bilateral basal ganglia are shown by the green arrows. An obvious hyperdensity is noted on the left side, as pointed by the arrow. No surrounding oedema or mass effect is present.

Non-contrast CT scan of her head (Figure 1) showed a hyperdense lesion in the left basal ganglia with no surrounding oedema or mass effect. It was done and reported on the day of admission. The report suggested that these changes could be due to metabolic abnormality rather than haemorrhage. This raised the suspicion of diabetic striatopathy. Accordingly, treatment was started with IV fluids and variable rate insulin infusion to address her hyperglycaemia and ketosis.

**Figure 2:**
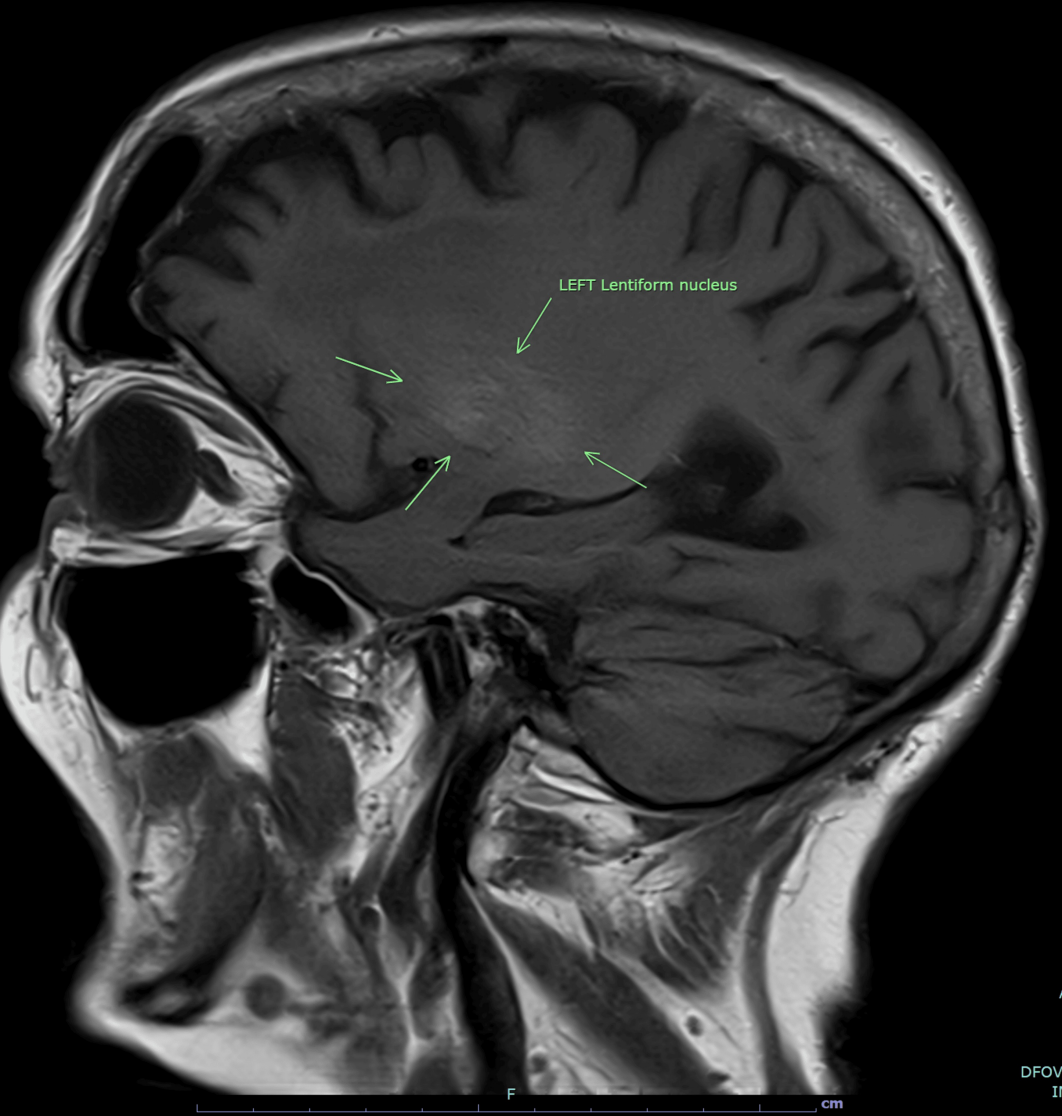
MRI brain T-1 parasagittal sequence image Slight hyperintensity noted in the area of the left lentiform nucleus, as pointed by the green arrows.

**Figure 3:**
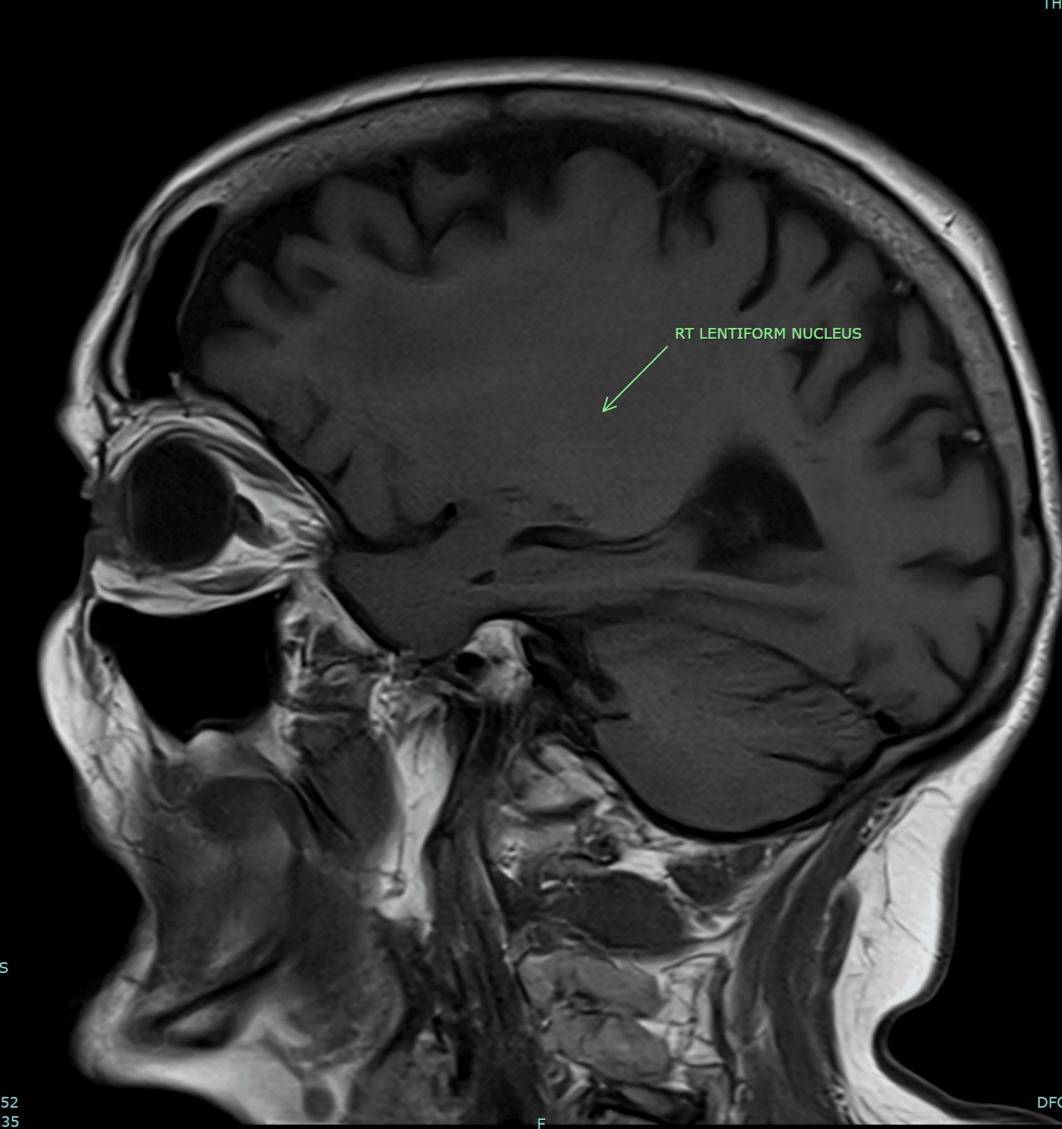
MRI brain T-1 parasagittal sequence image Area of the right lentiform nucleus is pointed out by the green arrow; no hyperintensity is noted.

She went on to have an MRI head the next day. It showed similar findings of slight hyperintensity (Figure 2) on the left side in the parasagittal T-1 sequence images. When compared on the parasagittal sequence images, no such hyperintensity was found on the right side (Figure 3). The MRI head ruled out any acute ischaemia.

Treatment with IV fluids and variable rate insulin infusion was continued. Her blood sugar and ketone levels started coming down to normal levels in about 24 hours. She was subsequently switched back to her original insulin regimen of Novomix-30 12 units and 6 units daily.

The choreoathetoid movement continued to persist even after correction of the metabolic abnormality. She was subsequently started on low-dose tetrabenazine, and the dose was gradually increased. There was marked improvement of her symptoms within one week of starting tetrabenazine.

She remained in the hospital for three weeks to facilitate optimal social care at her home after discharge. During this period, she was able to mobilise within the hospital with her walking frame. There was complete resolution of her choreoathetoid movements, and her blood sugar levels remained under control.

At her six-week follow-up with the diabetes team, her glycaemic control was better, and the movement disorder did not seem to recur. Tetrabenazine was continued.

## Discussion

Diabetic striatopathy is a rare complication of uncontrolled diabetes mellitus. It includes hyperglycaemia in association with hemichorea or hemiballismus, with or without a characteristic hyperdense or hyperintense lesion in CT or MRI scan, respectively [1-3].

Epidemiology

The true prevalence of this condition is unknown, but diabetic striatopathy is a rare and likely under-recognised condition [2]. Most cases have been reported in elderly patients (mean age 60-70 years) with a marked female predominance [1-4]. It has been more commonly reported in Asian populations [2,6].

Our patient was an 84-year-old lady from the United Kingdom. Her ethnicity and advanced age highlight the importance of vigilance in the elderly age group as well, more so because in the elderly age group, other comorbidities can obscure the diagnosis.

Pathogenesis

This condition was first reported in 1960 [5]. However, the pathogenesis is still not well understood [3,6,7]. Proposed mechanisms include impaired striatal glucose utilisation with resultant gamma-aminobutyric acid (GABA) depletion, microvascular ischemia, petechial haemorrhage and accumulation of metabolic by-products like calcium and manganese [3]. Positron emission tomography (PET) imaging has demonstrated hypometabolism in affected basal ganglia, suggesting a reversible metabolic dysfunction rather than irreversible infarction [2,9]. The most common finding noted in limited biopsy samples is that of gemistocytopathy [3]. Hyperglycaemia can lead to reduced cerebral blood flow, causing structural damage to the striatal astrocytes. These damaged astrocytes are called gemistocytes [3]. There can be further structural damage to the astrocytes, like haemorrhage, methaemoglobin deposition, mineral (calcium and manganese) deposition, myelinolysis and atrophy [3]. Another theory suggests that, in the setting of non-ketotic hyperglycaemia, the cerebral metabolism is shifted to the anaerobic pathway, leading to depletion of GABA [3,7]. Due to diminished GABA levels, there is a reduction in its inhibitory action in the basal ganglia, leading to hyperkinetic movements [7].

Interestingly, in the ketotic setting, GABA can be synthesised from acetoacetate [3]. As a result, hyperkinetic movements are less common in the ketotic setting [3]. However, in our case, the patient had ketotic hyperglycaemia.

Clinical features

Most patients present with unilateral movement disorders (hemichorea or hemiballismus) in the background of poorly controlled diabetes mellitus [1-4]. Chorea refers to involuntary, irregular, and non-rhythmic, brief, dance-like movements often involving the distal limbs. Ballismus is a severe, large-scale form of chorea characterised by violent flinging motion involving the proximal limbs. The most common presentation is that of unilateral arm and leg involvement [1]. Other variations may include the involvement of the face, peri-oral structures or bilateral chorea in very rare cases [1,3]. The movements typically worsen with movement or anxiety and improve with sleep or at rest [2]. Uncontrolled diabetes mellitus can present with other movement disorders, which can be hyperkinetic (chorea, dystonia, tremors, akathisia, restless leg syndrome) or hypokinetic (Parkinsonism) [1,3].

Our patient presented with the classical symptoms of hemichorea contralateral to the side of the radiological findings. The absence of any motor or sensory neurological deficit ruled out ischaemic or haemorrhagic stroke.

Investigations

This condition is more commonly seen in type 2 diabetes mellitus compared to type 1 [2]. The mean HbA1C in most literature reviews has been found to be markedly elevated, linking the pathogenesis with prolonged poor glycaemic control [1-4]. The mean value of blood sugar on presentation in the reported cases is markedly elevated [1-4,6], highlighting the role of acute hyperglycaemia in the background of poorly controlled diabetes. In the majority, blood and urinary ketones are found to be negative [2]. In rare cases, it can be the first presentation of diabetes mellitus [1-3].

Our patient was known to have type 2 diabetes mellitus. Her last HbA1C one year back was 73 mmol/L, indicating prolonged poor glycaemic control. Additionally, her blood ketone levels were raised at 3.5 mmol/L, which is quite unusual with diabetic striatopathy.

Neuroimaging is the cornerstone of the diagnosis of this condition. A CT scan shows hyperdensity of the striatum, on the opposite side of the movement disorder. T1-weighted MR images show hyperintensity of the striatum. The putamen is the most frequently involved structure, followed by the caudate nucleus; globus pallidus involvement is less common [1-4]. There may be discrepancies (mismatch or incompatibility) between CT and MRI findings (i.e., CT not showing abnormality, whereas MRI does, or showing different involvement), and in some cases, imaging changes and chorea onset are not synchronous [1-3]. In some cases, patients can present with a movement disorder but without radiological findings [3]. Therefore, it is very important to identify the clinical features promptly and start treatment.

Our patient had the classical radiological findings of hyperdensity of the left basal ganglia on CT (Figure 1) and slight hyperintensity of the left lentiform nucleus on the T-1-weighted MRI image (Figure 2). Absence of any surrounding oedema or mass effect helped to rule out haemorrhagic stroke.

Treatment

The mainstay of treatment is prompt correction of hyperglycaemia, which includes insulin therapy, rehydration, and correction of electrolyte abnormalities [1-3,7]. In the majority of cases, restoration of euglycemia alone is sufficient to control the symptoms [3]. However, many patients require additional medications targeting the chorea/ballism, such as antipsychotics (e.g. haloperidol), dopamine-depleting agents (e.g. tetrabenazine), benzodiazepines, valproate, or others [2,3].

In our case, the patient required the addition of tetrabenazine even after correction of hyperglycaemia and ketosis. The symptoms started improving after one week and were completely resolved by three weeks.

Prognosis

With prompt recognition and treatment, most patients have a favourable outcome with the initiation of treatment [1,3,10]. The changes in neuroimaging (CT and MRI) lag the clinical improvement and can take weeks to months to show resolution [2,3]. Delay in initiating treatment can sometimes lead to permanent structural changes and persistent deficits [10].

## Conclusions

This case highlights that diabetic striatopathy should be considered in elderly diabetic patients presenting with sudden-onset hemichorea, even when other comorbidities could confuse the diagnosis. Our patient’s presentation also shows that advanced age does not necessarily limit recovery if the condition is recognised early and managed appropriately. In addition, the persistence of movements despite correction of hyperglycaemia reinforces the need for symptomatic treatment, which, in this case, led to a good outcome. Overall, diabetic striatopathy remains an uncommon but important cause of acute movement disorders. Recognising its characteristic clinical and radiological features is essential to prevent misdiagnosis as stroke and to ensure timely treatment.
